# Loxhd1b inhibits the hair cell development in zebrafish: Possible relation to the BDNF/TrkB/ERK pathway

**DOI:** 10.3389/fncel.2022.1065309

**Published:** 2022-11-24

**Authors:** Jingwen Liu, Xu Zhang, Qingchen Zhang, Rongrong Wang, Jingyu Ma, Xiaohui Bai, Dawei Wang

**Affiliations:** ^1^Department of Clinical Laboratory, Shandong Provincial Hospital, Cheeloo College of Medicine, Shandong University, Jinan, China; ^2^Department of Ophthalmology, Jinan Second People’s Hospital, Jinan, China; ^3^Translational Medical Research Center, Wuxi No.2 People’s Hospital, Affiliated Wuxi Clinical College of Nantong University, Wuxi, China; ^4^Key Laboratory of Neuroregeneration of MOE, Nantong Laboratory of Development and Diseases, School of Life Sciences, Co-Innovation Center of Neuroregeneration, Nantong University, Nantong, China; ^5^Central Hospital Affiliated to Shandong First Medical University, Jinan, China; ^6^Department of Laboratory Medicine, Shandong Provincial Hospital Affiliated to Shandong First Medical University, Jinan, China; ^7^Department of Orthopedics, Shandong Provincial Hospital, Cheeloo College of Medicine, Shandong University, Jinan, China; ^8^Department of Orthopaedics, Shandong Provincial Hospital Affiliated to Shandong First Medical University, Jinan, China

**Keywords:** zebrafish, hair cell, Loxhd1b, BDNF/TrkB/ERK signaling pathway, morpholino

## Abstract

**Background:**

Mutations in lipoxygenase homology domain 1 (*LOXHD1*) cause autosomal recessive inheritance, leading to high-frequency and intermediate-frequency hearing losses in patients. To date, studies on the localization of *LOXHD1* gene expression are limited. In this study, we aimed to observe the expressions of *Loxhd1b* in zebrafish, C57BL/6 murine cochlea, and HEI-OC1 cells.

**Methods:**

The expression of *Loxhd1b* in the auditory system of zebrafish was explored by *in situ* hybridization experiments of zebrafish embryos. The expression of *Loxhd1b* in cochlear and HEI-OC1 cells of C57BL/6 mice was analyzed by immunofluorescence staining. Confocal microscopic in vivo imaging was used to detect the number and morphological characteristics of lateral line neuromasts and inner ear hair cells in zebrafish that knocked down *Loxhd1b* gene. The effect of knockdown *Loxhd1b* gene on the development of zebrafish otolith and semicircular canal was observed using microscopic. Transcriptome sequencing was used to identify downstream molecules and associated signaling pathways and validated by western blotting, immunostaining, and rescue experiments.

**Results:**

Results of the *in situ* hybridization with zebrafish embryos at different time points showed that *Loxhd1b* was expressed in zebrafish at the inner ear and olfactory pores, while the immunostaining showed that *Loxhd1* was expressed in both C57BL/6 mouse cochlea and HEI-OC1 cells. Loxhd1b knockdown causes a decrease in the number of spinal and lateral line neuromasts in the inner ear of zebrafish, accompanied by weakened hearing function, and also leads to developmental defects of otoliths and ear follicles. The results of transcriptomics analysis revealed the downstream molecule brain-derived neurotrophic factor (BDNF) and verified that *Loxhd1b* and BDNF regulate the formation of zebrafish hair cells by synergistic regulation of BDNF/TrkB/ERK pathway based on western blotting, immunostaining, and rescue experiments.

**Conclusion:**

This was the first time that the BDNF/TrkB/ERK pathway was identified to play a critical role in the molecular regulation of the development of zebrafish hair cells and the auditory development by *Loxhd1b*.

## Introduction

It has been documented that nearly 500 million people worldwide suffer from sensory impairment due to hearing loss (Wilson et al., 2017; Sheffield and Smith, 2018). Although many hearing related genes have been identified, Wei et al. (2022) it is important to understand the molecular mechanism regulating the hearing loss. Previous studies have shown that 60% of deaf patients are related to genetic factors (Bai et al., 2014). Based on the patient’s symptoms, genetic hearing losses are divided into two categories, i.e., syndromic (30%) and non-syndromic (70%) (Bai et al., 2014). In addition, there are four types of hereditary hearing loss based on the inheritance, including autosomal recessive inheritance, autosomal dominant inheritance, mitochondrial inheritance, and X-linked inheritance. The most common type of hereditary hearing loss is the autosomal recessive non-syndromic hearing loss (ARNSHL), which accounts for ∼80% of non-syndromic hearing loss (NSHL). However, there are many genes related to deafness, and the genetic methods used to investigate these genes are complex and diverse (Grillet et al., 2009; Pang et al., 2020). The explicit molecular functions of these genes in the formation of deafness largely remain unknown. Studying the pathogenesis of hereditary deafness is extremely challenging and new drugs to protect hearing are also crucial.

The *LOXHD1* gene is located in the 18q21.1 region of human chromosome 18, containing 48 exons and encoding the lipoxygenase homology domain containing protein 1 (LOXHD1) with a total of 2,068 amino acids (Pang et al., 2020). The LOXHD1 contains a total of 15 PLAT repeats. The *LOXHD1* gene is specifically expressed in the inner ear of mice (Grillet et al., 2009). It is first expressed in the nucleus of inner ear hair cells after birth, and then concentrated in the cytoplasm as the development progresses. At the mature stage of the mice, it is specifically expressed on the entire static surface of the cilia of the cochlear hair cells. The inner ear structure of mice with congenital deafness induced by ethylnitrosourea (ENU) appears abnormal, which is manifested as loss of hair cells and degeneration of spiral ganglion cells (Sweatt, 2001). *LOXHD1* plays an important role in the mechanical transduction of mature hair cells (Selcher et al., 2002). To date, studies on the expression and the effect of *Loxhd1b* on zebrafish’s auditory development are only limited to the use of mice but not verified by the use of zebrafish. Zebrafish have the advantage of studying hair cell development *in vivo*. Available data have shown that zebrafish shares approximately 70% of human genes, and more than 80% of known human disease-related genes have their counterparts in zebrafish (Kohashi and Oda, 2008). In comparison to the mouse model used in the previous studies of *Loxhd1b*, zebrafish is used as a biological model due to its visualization advantages, i.e., it is convenient to record and track the developmental process through imaging (Downes and Granato, 2004; Bandmann and Burton, 2010). Zebrafish are relatively genetically conserved with mice and human, by using which we can observe the auditory system *in vivo*. In particular, there are hair cells in the inner ear and lateral nerve mound, while the inner ear is located near the surface of the embryo, and there is a lateral nerve mound on the body surface, which is convenient for direct observation and intervention. In this regard, zebrafish has currently become a model organism for studying the development of ear vesicles and nerves that are closely related to human diseases (Leitner, 2014; MacRae and Peterson, 2015).

As a small protein isolated from pigs for the first time in 1982 (Bathina and Das, 2015), the brain-derived neurotrophic factor (BDNF) is the most widely distributed neurotrophic factor in the central nervous system (Segal, 2003; Cunha et al., 2010), mainly responsible for regulating the growth, development, differentiation, and repair of neurons, with an important regulatory effect on the structure and function of neurons. BDNF has also shown a protective effect on the hair cells and spiral ganglion cells of the inner ear of rodents (Liu et al., 2015). In the brain neurons, BDNF binds to two different receptors, including the high-affinity tyrosine kinase receptor B (Tropomyosin-related kinase B, TrkB) and the low-affinity p75 receptor (Fournier et al., 2012; Yan et al., 2017), to regulate the function of neurons. Studies have shown that in the hair cells of the inner ear and lateral nerve mounds of zebrafish, BDNF is involved in the survival and development of hair cells by binding to TrkB (Liu et al., 2015), while the BDNF can regulate the migration of zebrafish lateral line primordium and the maintenance of mechanoreceptor progenitor cells through the mediation of TrkB (Yi et al., 2014). In the brain neurons, the extracellular regulated protein kinase (ERK) is one of the important molecules of the MAPK family. The combination of BDNF and TrkB can activate the intracellular MAPK/ERK signaling pathway (Gao et al., 2020; Zhong et al., 2020), which plays an important role in regulating axon growth and neuron survival and differentiation. To date, no relationship between *Loxhd1b* and BDNF has been identified, and the function of the BDNF/TrkB/ERK pathway in the molecular regulation of zebrafish hair cell development and auditory development by *Loxhd1b* are still unclear.

Although the zebrafish model has been used extensively in the study of pathogenesis of a variety of medical disorders (Campbell et al., 2006; Kaiser et al., 2012; Huang et al., 2013; Leitner, 2014; MacRae and Peterson, 2015; Stawicki et al., 2015; Stewart et al., 2015; Coffin and Ramcharitar, 2016; Meshalkina et al., 2017; Hung et al., 2019; Yang et al., 2019; He et al., 2021), no study of *Loxhd1b* on zebrafish has been reported, and there is no mechanism of how *Loxhd1b* regulates hair cell development. In this study, the zebrafish model was used for the first time to reveal the molecular mechanism regulating the auditory development by *Loxhd1b*. Our results suggested that BDNF bound its specific receptor TrkB to activate the downstream molecule ERK, and the BDNF/TrkB/ERK pathway participated in the molecular regulation of auditory development by *Loxhd1b*.

## Materials and methods

### Animals

The zebrafish used in this study was kindly provided by Dr. Liu Dong from Nantong University (Nantong, China). Zebrafish wild-type AB line and Tg (brn3c: GFP) line was fed twice a day and kept in a fully automated zebrafish rearing system (ESEN, China; temperature 28 ± 0.5°C, pH 7.0, and conductivity 500 μS) with a photocycle of 14-h light and 10-h dark. Embryos were placed in 0.003% (w/v) 1-phenyl-2-thiourea (PTU) within 24 h to prevent pigmentation.

The suckling mice used in this study were postnatal 3 days old (P3) C57BL/6 mice. The P3 mice were purchased from the Animal Center of Shandong University (Jinan, China). After inhalation anesthesia, mice were acutely decapitated with dissecting scissors, and then quickly used for dissection and further experiments. Laboratory animal Use Permit [SYXK(LU) 2019 0005].

### Cell culture

The HEI-OC1 cells, a commonly used auditory cell line with the characteristics of hair cells, were used to investigate the molecular mechanism of auditory development (Gao et al., 2020; Zhong et al., 2020). The HEI-OC1 cells were cultured in the mixture of 5% volume of FBS without antibiotics and high-sugar DMEM (Gibco BRL, Grand Island, NY, USA) and kept in a 5% CO_2_ incubator at 33°C.

### Whole mount immunohistochemistry

The zebrafish immunohistochemistry experiments were performed based on the methods previously reported (Campbell et al., 2006). The antibodies used in this study included Alexa Fluor 488 antibody (1:1,000; Invitrogen), anti-myosin VIIa antibody (1:200; DSHB), Alexa’s anti-SOX2 antibody (1:500; abcam), and Fluor 594 secondary antibody (1:1,000; Invitrogen). Larvae were incubated with DAPI (1:500 dilution; Thermo Fisher Scientific) for 20 min to label the nucleus. Cells were fixed with 4% PFA for 30 min, then permeabilized with 1% Triton X-100 for 5 min and blocked with 1% BSA in phosphate buffered saline (PBS) for 1 h. Then, the cells were incubated with primary anti-Loxhd1 antibody (bs-18343R, Bioss), anti-BDNF antibody (orb318730, biorbyt), and anti-Myosin VIIa antibody (138-1, DSHB) at 4°C overnight, and then with both fluorescent secondary antibody and DAPI (D9542, Sigma) in dark for 1 h, and washed with PBS three times. The samples were observed under a laser scanning confocal microscope (Germany, LEICA).

### Whole mount *in situ* hybridization

The whole-body *in situ* hybridization (WISH) was performed based on the standard procedures (Nasevicius and Ekker, 2000; He et al., 2021). First, the *Loxhd1b* specific primers (forward primer 5′-GTGGTGGATGATGAGGAGATG-3′ and reverse primer 5′-TGTCCGACTCGATCACTCTG-3′) and the BDNF specific primers (forward primer 5′-AGGAGTTGCTTGAGGTGGAA-3′ and reverse primer 5′-TATCCATGGTAAGGGCTCGC-3′) were used to amplify the target fragments from zebrafish cDNA and transform into the pGEM-T Easy vector. Then, the DIG RNA labeling kit was used to transcribe the gene-specific digoxin-labeled RNA probe *in vitro*. The pre-immobilized embryos were incubated with the probe overnight at 4°C. Second, an alkaline phosphatase (AP)-conjugated anti-digoxigenin (Roche, #11093274910) antibody was used to detect the digoxin-labeled RNA probe. The samples were observed with pictures taken with the stereo microscope MVX10 (Japan, Olympus).

### Morpholino and mRNA injection

Studies have shown the successful method used to knock down targeted genes in zebrafish using morpholino (MO) (Summerton and Weller, 1997; Summerton, 1999; Leitner, 2014). Meanwhile, the MO-mediated gene downregulation method has been well applied in *Xenopus* and zebrafish embryos (Summerton and Weller, 1997; Summerton, 1999). However, many limitations in the application of MO are revealed, i.e., the downregulation of gene expression is not achieved through stable genetic manipulation, causing unstable efficiency with the relatively short maintenance time, only about 3 days (Summerton, 1999; Bill et al., 2009). Gene Tools was used to synthesize *Loxhd1b* MO (5′-CAGTGATGCTGAAAGTCTGACTTGC-3′). Embryos obtained from natural mating of Tg (Brn3c: GFP) zebrafish were used for microinjection. The MO was diluted with RNase-free water and injected into embryos at single-cell stage, which were then cultured in egg water medium at 28.5^°^C.

### FM1-43FX staining

Zebrafish hair cells were labeled with viability dye FM1-43FX (Molecular Probes, Invitrogen), which was a fixable analog of N-(3-triethylammonium propyl)-4-(4-(dibutyl) (Amino)-styryl) pyridinium dibromide (FM1-43). FM1-43FX was thought to mark hair cells by traversing mechanically sensitive channels (Liu et al., 2015; Yan et al., 2017). In this experiment, in order to label the hair cells in neuroma, the larvae were placed in embryo medium containing 0.3 μg/ml FM1-43FX for 30 s, and washed 3 times in fresh embryo medium. Finally, the fish was anesthetized with 0.02% Tricaine (MS-222; Sigma) to observe the hair cells.

### Western blot analysis

The HEI-OC1 cells were cultured in a 100 mm cell culture dish, and after the treatment of RNA interference, the cells were collected by centrifugation. Then, PMSF was added to the cytoplasmic protein lysate, and the cells were transferred into 0.22 μm PVDF and incubated with primary antibody BDNF (ab108319; abcam), anti-TrkB (bs-3732R; bioss), anti-ERK (12754S; CST), anti-β-actin (ab8226; abcam) diluted 1:1000 (4°C, overnight), and anti-rabbit IgG secondary antibody (ZB-2301; zsbio) at 25°C for 1.5 h. After washing, the immune complexes were detected using a chemiluminescence imaging system (model: Al480; Bio-Rad).

### mRNA sequencing

The total RNA of the samples between Loxhd1b knock-down and wild type zebrafish were extracted with TRIzol reagent (Invitrogen) and analyzed by Agilent 2100 Bioanalyzer (Agilent Technologies, USA). The Illumina HiSeq platform was used to prepare and sequence libraries according to the manufacturer’s instructions (Illumina, USA). The sequences were processed and analyzed by GENEWIZ with the *p*-value set to < 0.05 to reveal differentially expressed genes (DEGs).

### RNA isolation and real-time PCR

Total RNA was extracted from zebrafish embryos by TRIzol reagent according to the manufacturer’s instructions (Thermo Fisher Scientific, USA) with the genomic contaminations eliminated by DNaseI. Total RNA (2 μg) was reversely transcribed using a reversed first strand cDNA synthesis kit (Fermentas, USA) and stored at –20^°^C. The sequences of PCR primers used for validating the splice-blocking MO were forward primer 5′-GAAAGCGGAGAAGGAGGACT-3′ and reverse primer 5′-CAGCCGTGAACACACTCACT-3′. The housekeeping gene *ef1a* amplified with forward primer 5′-CTTCAACGCTCAGGTCATCA-3′ and reverse primer 5′-CGGTCGATCTTCTCCTTGAG-3′ was used as internal control to produce a 126-bp amplification product.

### C-startle response test

The hearing and balance functions of zebrafish are mainly related to the development of the inner ear (De Rose and Farber, 2003; Huang et al., 2013). We performed a fast escape reflex and used two near-field pure tone stimuli with different sound intensity levels to perform hearing tests on MO-injected zebrafish (Kohashi and Oda, 2008). This experiment was tested in a 96-well plastic plate and recorded with a high-speed camera (Redlake, MotionScope M3, 1000 fps) under infrared light illumination. Larvae in each group was tested 15–18 times with the movement distance after the startled reaction calculated.

### Statistical analysis

All data were analyzed using GraphPad Prism 8.0.2. Statistical analysis was performed by one-way analysis of variance (ANOVA) and Student’s *t*-test or Chi-square test with the *p*-value < 0.05 considered statistically significant. Each experiment was repeated with 3 or more biological replicates.

## Results

### *Loxhd1b* was expressed in zebrafish ear vesicles and olfactory pores

**FIGURE 1:**
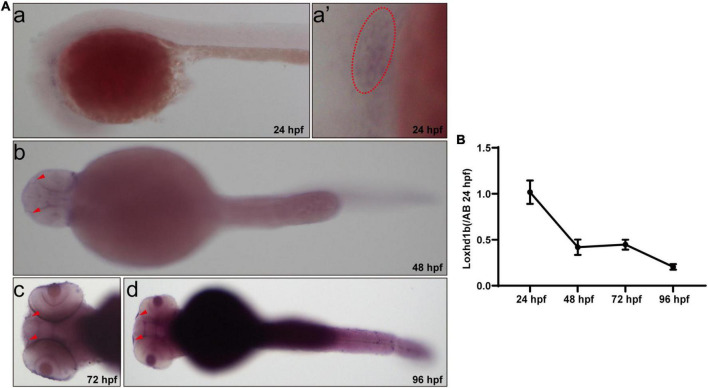
Expression of *Loxhd1b* in zebrafish. **(A)** *In situ* hybridization revealing the *Loxhd1b* expression around the otic vesicles and Olfactory hole, indicating its contribution to hearing development. The arrows indicate the olfactory hole. The ellipse of dashed line indicates the otic vesicle. **(B)** *Loxhd1b* expression gradually decreased from 24 to 96 hpf.

Initially, the results of WISH experiments showed that the expression of *Loxhd1b* in zebrafish was detected by digoxigenin-labeled *Loxhd1b* probe (Figure 1). The *Loxhd1b* gene was highly conserved and was expressed in zebrafish ear vesicles at 24 h post-fertilization (hpf) and over time, was also detected in the olfactory pores (Figure 1A). In addition, the results of real-time PCR (RT-PCR) (Figure 1B) showed that the expression of *Loxhd1b* was gradually decreased from 24 to 96 h over time, which were consistent with the results by the *in situ* hybridization.

### Deficiency of *Loxhd1b* caused decreased hair cells and neuromasts

**FIGURE 2:**
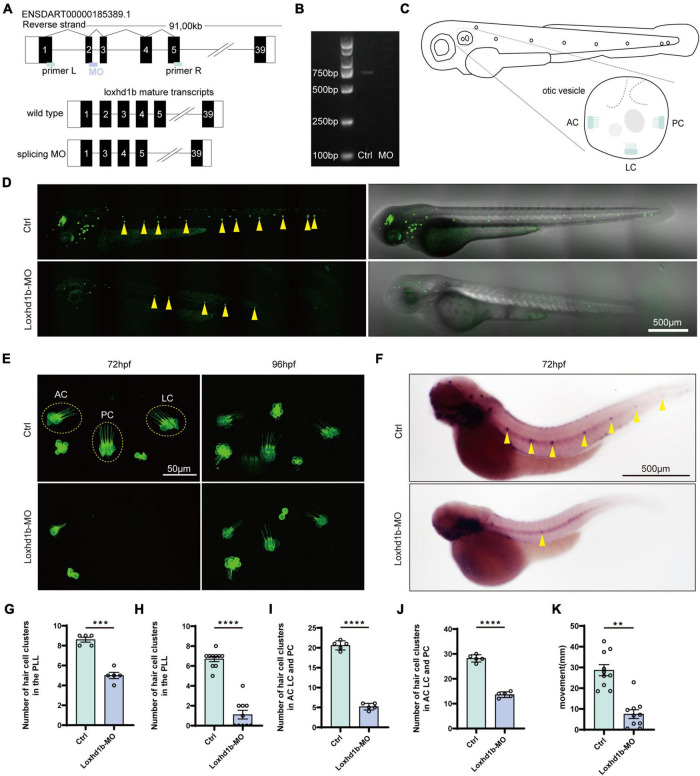
Effect of *Loxhd1b* knockdown on decreasing the number of hair cells in the otic vesicle and posterior lateral line of zebrafish. **(A)** The targeting site of *Loxhd1b* splice blocking morpholino and the PCR primer design for validating the knockdown analysis. The wild type mature transcripts indicate the natural splicing product of *Loxhd1b* mRNA. The splicing MO mature transcripts indicate the abnormal splicing product of *Loxhd1b* mRNA with Exon 2 deletion caused by morpholino injection. **(B)** Effectiveness of *Loxhd1b* knockdown confirmed by PCR. **(C)** The schematic for three clusters of cristae hair cells in the otic vesicle. ACHC, anterior cristae hair cells; LCHC, lateral cristae hair cells; PCHC, posterior cristae hair cells. **(D)** Fluorescence microscopic imaging analysis of *Loxhd1b* knockdown line at 3 dpf. Arrowheads indicate hair cell clusters. Scale bar = 500 μm. **(E)** Confocal imaging analysis of cristae hair cells in the otic vesicle of control and *Loxhd1b* deficiency zebrafish at 72 and 96 hpf. Scale bar = 50 μm. **(F)** WISH experiments of the eya1 gene and the imaging analysis of control, claudin h morphants, and rescued zebrafish at 96 hpf in bright field. Scale bar = 500 μm. **(G)** Statistical analysis of zebrafish lateral line neuromast at 72 hpf. **(H)** Statistics of zebrafish lateral line neuromasts at 96 hpf. **(I)** Statistical analysis of the number of different cristae hair cells in the inner ear of control and *Loxhd1b*-MO in 72 hpf. **(J)** Statistical analysis of the number of different cristae hair cells in the inner ear of control and Loxhd1b-MO in 96 hpf. **(K)** C-startle response in *Loxhd1b* morphants zebrafish larvae was significantly lower than that in control zebrafish. **P < 0.01, ***P < 0.001, and ****P < 0.0001.

Results of gene knockdown analysis showed that MO effectively affected the splicing of *Loxhd1b* precursor mRNA, resulting in deletion of exon 2 and reading frame shifted (Figures 2A,B). To further investigate the status of hair cells, we observed and counted the number of three inner ear hair cell clusters, i.e., the anterior crest (AC), posterior crista (PC), and lateral crest (LC) (Figure 2C). By using genetically modified fish, we visually evaluated the changes in zebrafish inner ear hair cells after the *Loxhd1b* gene knockdown. The confocal results showed that 3 days after the injection of *Loxhd1b* MO, the number of hair cells in each nerve cluster in the lateral line of zebrafish was significantly reduced (Figures 2D,G). In the 72-hpf zebrafish inner ear hair cells, we counted the total number of hair cell clusters of morphant (15) and WT (32) (Figures 2E,I). We also counted the total number of hair cell clusters in the inner ear of zebrafish at 96-hpf of MO (15) and Ctrl (32) (Figures 2E,J). Since the hair cells on the lateral line play a vital role in sensing changes in the surrounding environment, therefore, we further performed WISH experiments based on eya1 specific probes (Figures 2F,H). The results showed that the *Loxhd1b* gene knockdown caused decreased number of neuromas in zebrafish. Furthermore, we designed a C-shaped startle response to explore the effect of *Loxhd1b* on zebrafish hearing. Results showed that the movement distance of *Loxhd1b*-morphant zebrafish C-startle response was significantly lower than that of WT zebrafish (Figure 2K).

**FIGURE 3:**
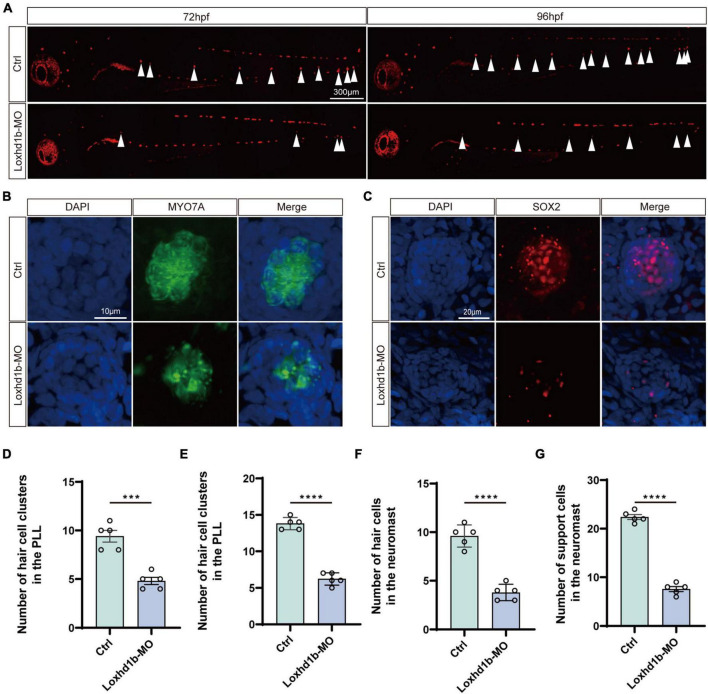
Morphology and number of hair cells and support cells in *Loxhd1b*-knockdown and control zebrafish. **(A)** Reduced numbers of neuromast hair cells in *Loxhd1b*-morphant fish labeled by FM1-43FX at 72 and 96 hpf. The arrows point to the position of posterior lateral line neuromasts. Scale bar = 300 μm. **(B,C)** Immunostaining of zebrafish lateral line hair cells and support cells at 72 hpf. Hair cells are stained with myosin-7a antibody and support cells were stained with SOX2 antibody. Scale bar = 10 and 20 μm. **(D–G)** Statistical analysis of the hair cell clusters in control and *Loxhd1b*-morphan based on *t*-test. ***P < 0.001 and ****P < 0.0001.

Because the sensory cells of the lateral neuromound are composed of hair cells and supporting cells, we then explored the effect of the knockdown of *Loxhd1b* in zebrafish on the formation and maturation of hair cells and supporting cells in lateral neuroma. The results showed that the number of hair cells in the lateral line neuroma labeled with FM1-43FX were significantly lower in *Loxhd1b*-morphant zebrafish than that in the WT group (Figures 3A,D,E). Meanwhile, we performed the immunohistochemical experiments to label Sertoli cells and hair cells using SOX2 antibody and myosin-7a antibody. The results showed that the number of hair cells in *Loxhd1b*-morphant zebrafish lateral line neuroma was significantly reduced (Figures 3B,F) as well as the number of support cells (Figures 3C,G).

### Deficiency of *Loxhd1b* caused dysplasia of hair cells not due to apoptosis of hair cells but decreased proliferation of support cells

**FIGURE 4:**
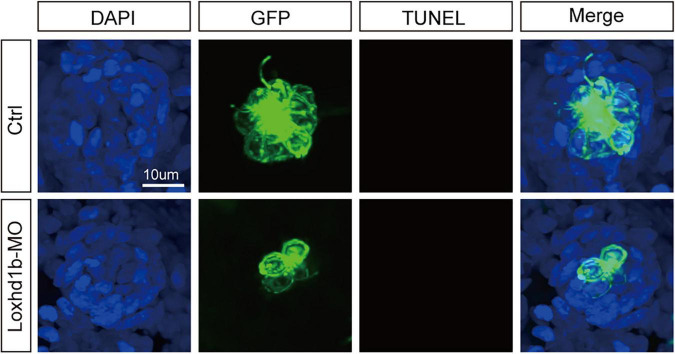
TUNEL cell apoptosis experiment in 72 dpf *Loxhd1b* knockdown zebrafish, showing that no apoptosis signal is detected. Scale bar = 10 μm.

To identify the factors that cause the reduction of zebrafish inner ear and lateral line hair cells, previous studies have reported that the loss of hair cells may be caused by the apoptosis of hair cells (Chen et al., 2003; Slattery and Warchol, 2010; Liu et al., 2016; Wang et al., 2017) with the TUNEL test (Stooke-Vaughan et al., 2015; Wang et al., 2017; Li et al., 2018; Yang et al., 2019; Zhang et al., 2019) for diagnosis of apoptosis; alternatively, the loss of hair cells may be due to the abnormal proliferation of support cells, while the hair cells were differentiated from support cells. Therefore, we used TUNEL to test the *Loxhd1b* knockdown zebrafish model, and found no TUNEL-positive cells, indicating no apoptosis of hair cells (Figure 4). Further studies showed that the abnormal function of *Loxhd1b* caused the hair cell dysplasia was not due to apoptosis of hair cells but by reduced proliferation of support cell.

### Deficiency of *Loxhd1b* caused developmental defects of otic vesicle and otoliths

**FIGURE 5:**
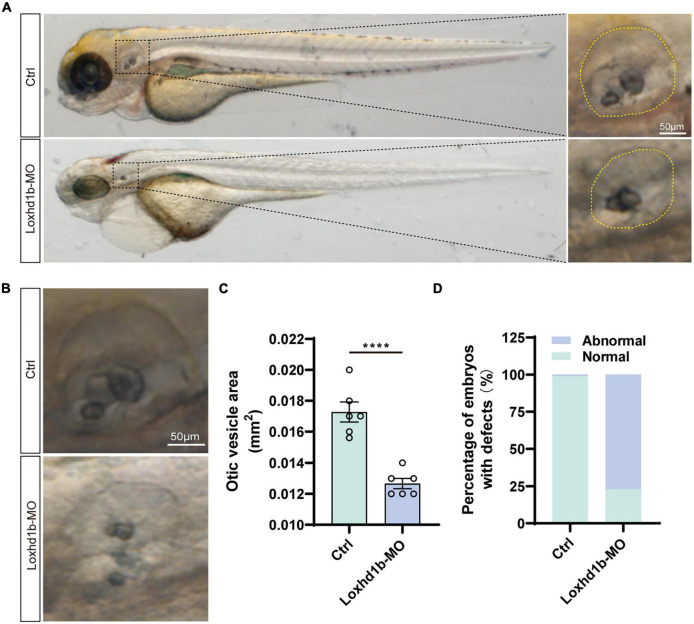
Loss of function of *Loxhd1b* causes the defects of otic vesicle and otoliths. **(A,B)** Imaging analysis of otic vesicle in control and *Loxhd1b* knockdown at 72 hpf. The yellow dotted line marks the boundary of the otic vesicle. Scale bar = 50 μm. **(C)** Statistical analysis of otic vesicle area in the control and *Loxhd1b* morphants at 72 hpf. **(D)** The *Loxhd1b* morphants with the utricle otolith lost, showing the decrease in number and defects in shape of otoliths, i.e., the *Loxhd1b* morphants showing either the utricle otolith lost or an abnormal saccular otolith. ****P < 0.0001.

It was reported previously that the abnormal development of otoliths led to balance disorders (Stooke-Vaughan et al., 2015). As pre-*in situ* hybridization showed significant expression of *Loxhd1b* in the ear vesicles, we further used confocal microscopy to observe the morphological development of ear vesicles and otoliths in zebrafish at 72 hpf. The results showed that the size of the 72 and 96 hpf ear capsules of *Loxhd1b* morphant was significantly smaller than the control group (Figures 5A,C). In addition, compared with the control group, the number and shape of otoliths in 77% of *Loxhd1b* morphant showed a significant reduction or defect (Figures 5B,D).

### *Loxhd1b* regulated TrkB/ERK signaling pathway to cause auditory abnormalities

**FIGURE 6:**
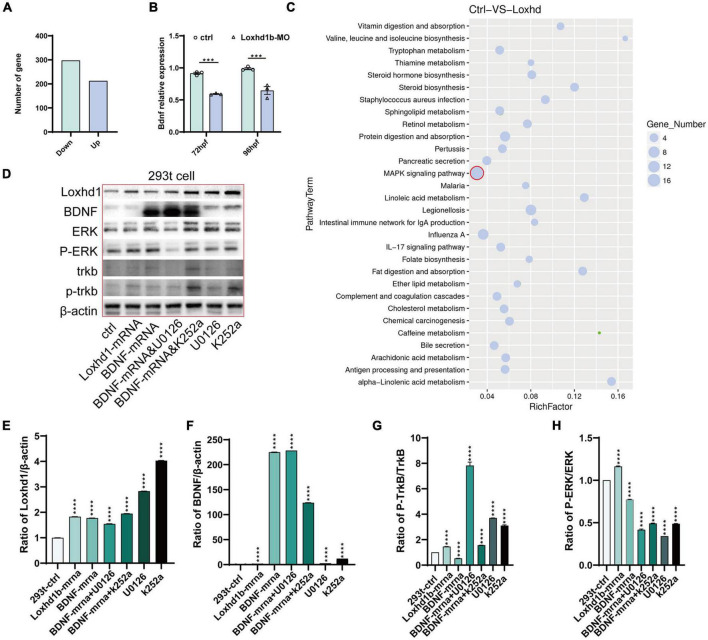
BDNF/TrkB/ERK signaling pathway regulated by the *Loxhd1b* gene in zebrafish auditory development. **(A)** Clustering analysis showing the replicates within group with a sound repeatability, while the control and mutated group are different in the clustering. **(B)** Volcano diagram of differentially expressed genes. Red and blue dots indicate up-regulated and down-regulated genes, respectively. Abscissa indicates gene fold change in different samples; ordinate represents statistical significance of gene expression change. **(C)** KEGG analysis of the differentially expressed genes. The vertical and horizontal axes represent the pathways and the Rich factor, respectively. The size of the dot indicates proportionally the number of differentially expressed genes in the pathway, and the color of the dot corresponds to a different *Q* value range. **(D–H)** Detection of protein expression of *Loxhd1b*, BDNF, TrkB, P-TrkB, ERK, and P-ERK based on chemiluminescence imaging system. ***P < 0.001 and ****P < 0.0001.

Transcriptome sequencing was performed based on the zebrafish embryos injected with *Loxhd1b*-morphant at 120 hpf and the control group. The results showed that compared with the control group, a total of 212 and 297 genes were significantly up- and down-regulated in *Loxhd1b*-morphant, respectively (Figure 6A). These results were further verified by real-time PCR analysis to reveal the difference between the BDNF gene at 72 and 96 hpf *Loxhd1b*-morphant and the control group, respectively (Figure 6B). The results of the enrichment analysis based on the Kyoto Encyclopedia of Genes and Genomes (KEGG) database (He et al., 2021) of these DEGs revealed a total of 30 metabolic pathways enriched, e.g., the MAPK signaling pathway (Figure 6C). In order to explore the relationship between the BDNF/TrkB/ERK signaling pathway and the effect of *Loxhd1b* on auditory abnormalities, we used the TrkB inhibitor K252a and the ERK inhibitor UO126 to act on the 293T cells. The results of western blot showed that BDNF regulated the activation of its specific receptor TrkB, i.e., the overexpression of BDNF gene enhanced the activation of TrkB, which was required to mediate BDNF’s involvement in *Loxhd1b* regulation of auditory development, whereas BDNF could not rescue *Loxhd1b* expression after TrkB was blocked. The results of western blotting experiments further showed that BDNF could regulate the activation of ERK, i.e., the overexpression of BDNF enhanced the activation of ERK, which was required for the regulation of auditory development by Loxhd1b. After the ERK was blocked, BDNF could not rescue the expression of Loxhd1b (Figures 6D–H). These results suggested that the regulation of Loxhd1b expression by BDNF blocked the TrkB/ERK signaling pathway, causing the auditory abnormalities, while Loxhd1b did not affect BDNF expression.

### BDNF and *Loxhd1b* were expressed in HEI-OC1 cell line and cochlea of C57BL/6 mice

**FIGURE 7:**
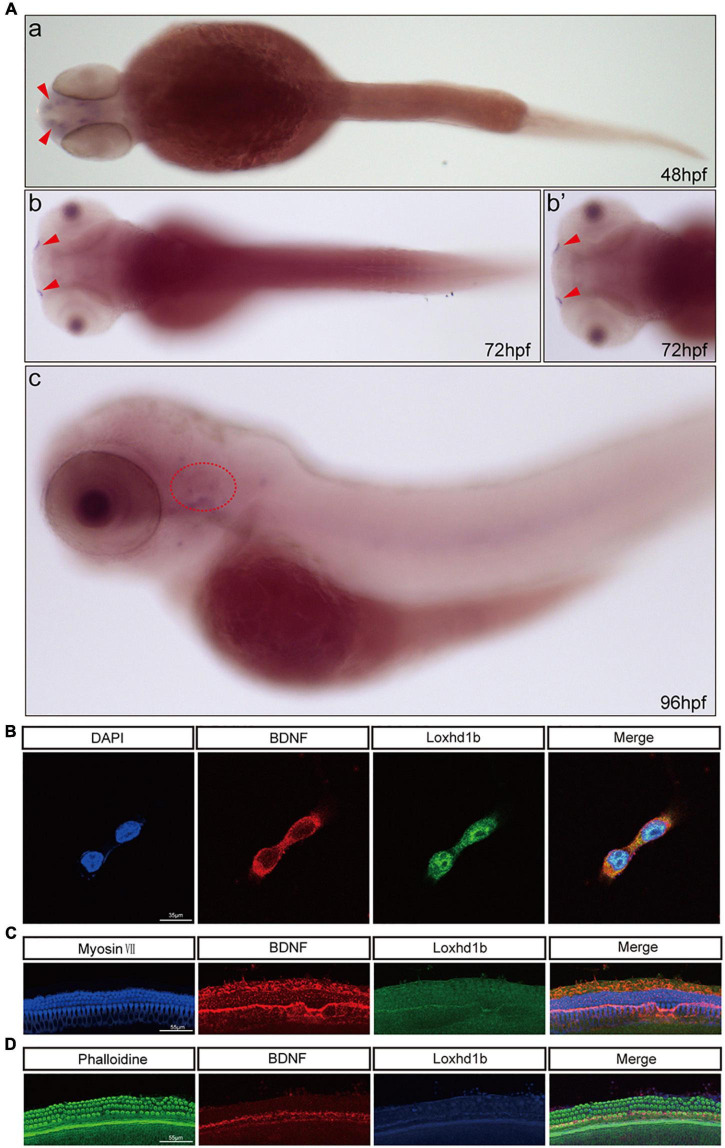
Co-localization of *Loxhd1b* and BDNF. **(A)** *In situ* hybridization at 48–96 hpf showing that BDNF is expressed around the inner ear and olfactory pore of zebrafish, suggesting the effect of BDNF on hearing development. The red arrow indicates the olfactory pore of the zebrafish, and the red dotted circle indicates the inner ear of the zebrafish. **(B)** Immunofluorescence staining of *Loxhd1b* and BDNF in HEI-OC1 cells. The nuclei are stained blue by DAPI. Scale bar = 35 μm. **(C)** Representative images of inner ear hair cells in cochleae from C57 mice of 7 days old. The inner ear hair cells are labeled with Myosin VII (blue). Scale bar = 55 μm. **(D)** Representative images of inner ear hair cells in cochleae from p57 mice of 7 days old. Phalloidin is used to label the inner ear hair cells (green). Scale bar = 55 μm.

In order to further determine the relationship between BDNF and *Loxhd1b*, we first explored the expression location of BDNF in zebrafish tissue by whole embryo *in situ* hybridization. The results were consistent with those of Loxhd1b, i.e., both BDNF and *Loxhd1b* were expressed in the inner ear and olfactory pore of zebrafish (Figure 7A).

We also detect the localization of both *Loxhd1b* and BDNF by immunofluorescence analysis with the results observed under confocal microscopy, showing that *Loxhd1b* and BDNF proteins were highly enriched in hair cell cytosol and slightly distributed in nucleus (Figure 7B). Next, we used 3-day old C57BL/6 mouse basement membrane for immunostaining and specific immunostaining with myosin VII and Phalloidin. The observation under a confocal microscope showed that *Loxhd1b* and BDNF were specifically expressed in inner and outer hair cells (Figures 7C,D).

### BDNF mRNA injection rescued the phenotype induced by zebrafish *Loxhd1b* gene defect

**FIGURE 8:**
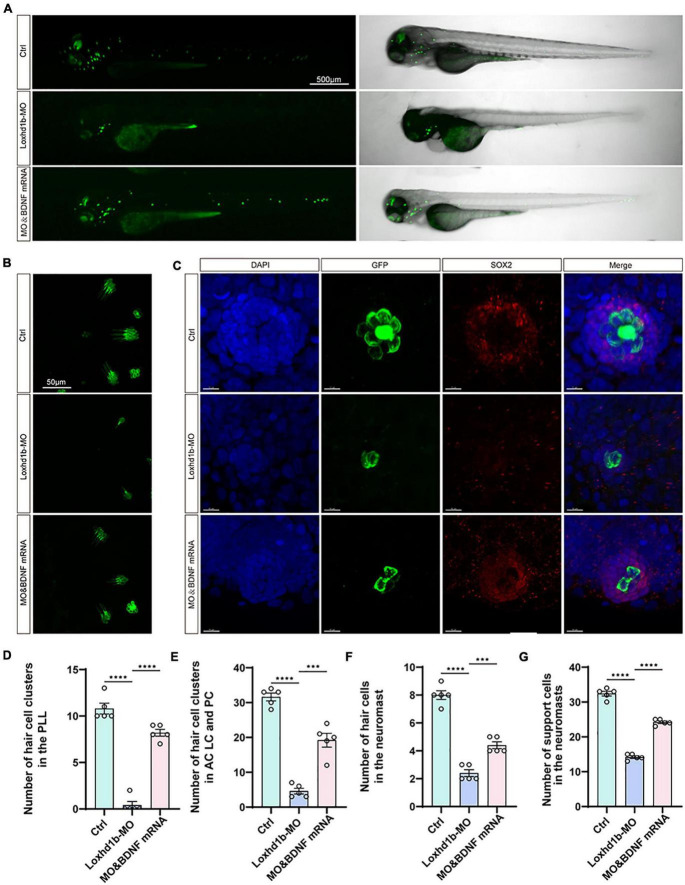
The developmental defects of hair cells in *Loxhd1b* morphants rescued by BDNF mRNA. **(A)** Imaging analysis of control, *Loxhd1b* morphants, and rescued zebrafish at 72 hpf in bright field (right) and fluorescent field (left). Scale bar = 500 μm. **(B)** Imaging analysis of cristae hair cells in control, *Loxhd1b* morphants, and rescue group at 72 hpf. Scale bar = 50 μm. **(C)** Imaging analysis of support cells in control, *Loxhd1b* morphants, and rescue group at 72 hpf. Scale bar = 50 μm. **(D)** Quantification of the number of hair cell clusters in the posterior lateral line of different groups at 72 hpf. **(E–G)** Statistical analysis of the number of different hair cells and support cells in different groups at 72 hpf. ***P < 0.001 and ****P < 0.0001.

**FIGURE 9:**
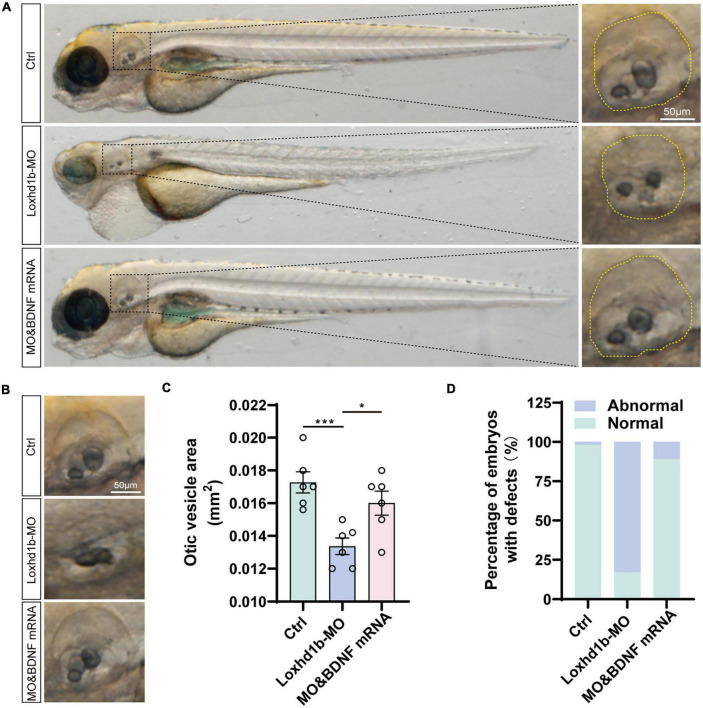
Loss of function of *Loxhd1b* causing the defects of otic vesicle and otoliths. **(A)** Imaging analysis of otic vesicle and cristae hair cells in control, *Loxhd1b* morphants, and rescue group at 96 hpf. The yellow dotted line marks the boundary of the otic vesicle. Scale bar = 50 μm. **(B)** BDNF mRNA is effective to rescue the reduced number of otoliths and smaller size of otic vesicle caused by *Loxhd1b* MO. Scale bar = 50 μm. **(C)** Statistical analysis of otic vesicle area in different groups at 96 hpf. **(D)** Proportions of embryos with developmental defects at 72 dpf. *P < 0.05 and ***P < 0.001.

In order to verify the rescuing effect of the exogenous BDNF injection on the absent phenotype of hair cell development in *Loxhd1b* morphant, we injected both the synthetic BDNF mRNA and *Loxhd1b* MO into zebrafish embryos at the single-cell stage *in vitro*, and performed confocal observations in 3 days. Results showed that the number of hair cell clusters in the ear follicle and lateral line of *Loxhd1b* morphant were partially restored (Figures 8A–G). Similarly, compared with *Loxhd1b* morphant, co-injection of BDNF mRNA also partially rescued abnormal phenotypes, including smaller ear follicle size and a decrease in the number of hair cells in the ear follicle (Figures 9A–D).

## Discussion

A growing body of evidence suggests that zebrafish hair cells (HCs) have similar morphology and functions to those in mammals in transmitting sensory information. Therefore, zebrafish lateral line neuroma has become a model for studying factors affecting the development and function of HCs. To date, however, no studies on the effect of *Loxhd1b* on HCs in zebrafish animal models have been reported in literature. Our study, for the first time, successfully constructed a *Loxhd1b*-deficient zebrafish model for studying the coordinated control of hair cells at the molecular level.

It has been reported that in the inner ear of *Loxhd1*Sba/Sba mice, *Loxhd1* mRNA is selectively expressed in hair cells, while the protein encoded by *Loxhd1* is located in the hair strands (Pang et al., 2020). However, the *Loxhd1* expression in zebrafish is unknown. In our study, the results of both *in situ* hybridization and RT-PCR showed that *Loxhd1b* was expressed in zebrafish ear vesicles and olfactory pores. The specificity of the expression site suggested that the *Loxhd1b* gene may affect the development and function of the sensory and balance systems in zebrafish. These results were consistent with those derived from the HEI-OC1 cell line and C57BL/6 mice, indicating that *Loxhd1b* played an important role in the development of hair cell nerve mounds.

In order to simulate the loss of function of *Loxhd1b*, we performed the *Loxhd1b* knockdown experiment in zebrafish using MO. Our results showed that compared with WT, the number of neuromas along the body was reduced and the neuroma lacked hair cells. *Loxhd1b* was also expressed in the hair cells of the inner ear, showing a temporal expression pattern, indicating that the inner ear and lateral line nerve mounds of the zebrafish were reduced within 3 days. The results of startle response showed that the *Loxhd1b*-morphant zebrafish swimming behavior was abnormal, indicating the effect of *Loxhd1b* on the auditory development. These results were consistent with those reported previously, showing that *Loxhd1*Sba/Sba mice containing PLAT10 repeat missense mutations exhibited hearing loss at 3 weeks of age (Pang et al., 2020). Furthermore, it was reported for the first that our results revealed significant morphological changes in the otolith size and semicircular canal between *Loxhd1b*-morphant zebrafish in comparison with the control, suggesting that *Loxhd1b* also played an important role in the balance function of zebrafish.

In general, medium and high frequency sensorineural hearing loss without vestibular disease will lead to stable or progressive hearing loss caused by *Loxhd1b* (Sweatt, 2001; Selcher et al., 2002; Kohashi and Oda, 2008). The severity of hearing loss varies from mild to profound. However, studies on the *Loxhd1* mutations associated with ARNSHL are limited with no functional experiments performed to confirm the pathogenesis of these mutations. Studies have shown that the BDNF gene was down-regulated and closely related to the development of spiral ganglion neurons (Wyllie, 1980; Das and Rajanikant, 2014; Sloan-Heggen et al., 2016; Gong et al., 2017; Zhang et al., 2017; Bai et al., 2020; Germana et al., 2020; Marchetta et al., 2020; Kempfle et al., 2021; Vink et al., 2021). In this work, our results of transcriptome sequencing after the injection of MO showed that there were 509 DEGS (212 up-regulated and 297 down-regulated) in zebrafish after the *Loxhd1b* knockdown. Notably, the expression of BDNF mRNA was down-regulated by up to 4.9 times in comparison with WT. These results suggested that BDNF was probably a downstream molecule of *Loxhd1b*, ultimately causing the abnormal auditory development of *Loxhd1b*. Furthermore, the results of *in situ* hybridization and immunostaining showed that BDNF and *Loxhd1b* were co-expressed in hair cells, which was consistent with the results reported previously, showing that the BDNF system existed in the inner ear of zebrafish (Germana et al., 2020). Moreover, the injection of the BDNF mRNA into the 1-cell stage of zebrafish rescued the loss of hair cells and the development of otolith semicircular canals, suggesting that the BDNF may be a downstream molecule of *Loxhd1b.* Overall, these results demonstrated the novel role of *Loxhd1b* in both auditory development and balance maintenance in zebrafish.

Lastly, we further explored the molecular mechanism regulating the auditory development by *Loxhd1b*. Previous studies have shown that BDNF regulates synaptic function by either activating a series of downstream signal pathways, including mitogen extracellular signal-regulated kinase/extracellular signal-regulated protein kinase (MEK/ERK) signal pathway, or through TrkB (Segal, 2003; Downes and Granato, 2004; Kohashi and Oda, 2008; Bandmann and Burton, 2010; Cunha et al., 2010; Leitner, 2014; Bathina and Das, 2015; Liu et al., 2015; MacRae and Peterson, 2015). In the current investigation, it’s the first time we find that the abnormal expression of Loxhd1b regulates BDNF, which can block the TrkB/ERK signaling pathway and cause auditory disorders; at the same time, BDNF positively regulates *Loxhd1b*. Therefore, we infer that the *Loxhd1b* gene is closely related to zebrafish hearing, which in turn, provided the potential prevention and treatment of patients with hearing loss in the future.

In summary, our study showed that *Loxhd1b* played important roles in the zebrafish auditory development, which is closely related to the effects of BDNF receptor and its downstream molecules. It was concluded that BDNF was of great significance for further exploring the treatment of hereditary deafness, providing strong experimental foundation for future development of novel drug and gene therapy.

## Data availability statement

The datasets presented in this study can be found in online repositories. The names of the repository/repositories and accession number(s) can be found below: NCBI Sequence Read Archive (SRA): SRR22207161, SRR22207162, SRR22207163, SRR22207164, SRR22207165, and SRR22207166.

## Ethics statement

The animal study was reviewed and approved by the Administration Committee of Experimental Animals, Jiangsu Province, China.

## Author contributions

DW and XB supervised and designed the project. JL, QZ, and XZ wrote the manuscript. JL, QZ, XZ, and RW analyzed the data. JL, QZ, XZ, RW, and JM performed the experiments. All authors contributed to the article and approved the submitted version.
